# Supplementary material for: The genome sequence of a tachinid fly, Linnaemya tessellans (Robineau-Desvoidy, 1830)
Source: Wellcome Open Res. 2024 May 9;9:243. [Version 1] doi: 10.12688/wellcomeopenres.21499.1 (PMC11310650; doi:10.12688/wellcomeopenres.21499.1)
Supplement: Peer review history

# Reviewer report: Reviewer response for version 1

**DOI:** 10.21956/wellcomeopenres.23769.r87126

**Published:** 2024-08-08

**Authors:** Bingmiao Gao

**Affiliations:** 1 School of Pharmacy, Hainan Medical University, Haikou, China

The author presented a genome assembly from one male *Linnaemya tessellans* (Tachinid fly; Arthropoda; Insecta; Diptera; Tachinidae). The genome sequence is 709.9 megabases in span. Most of the assembly is scaffolded into 7 chromosomal pseudomolecules, including the X and Y sex chromosomes. The mitochondrial genome has also been

 assembled and is 17.24 kilobases in length. However, to keep readers interested, I believe the author can provide more comparative analysis with current related species and draw a conclusion.

Are sufficient details of methods and materials provided to allow replication by others?

Yes

Is the rationale for creating the dataset(s) clearly described?

Yes

Are the datasets clearly presented in a useable and accessible format?

Yes

Are the protocols appropriate and is the work technically sound?

Yes

Reviewer Expertise:

Multi omics

I confirm that I have read this submission and believe that I have an appropriate level of expertise to confirm that it is of an acceptable scientific standard.

# Reviewer report: Reviewer response for version 1

**DOI:** 10.21956/wellcomeopenres.23769.r88491

**Published:** 2024-07-16

**Authors:** James B. Whitfield

**Affiliations:** 1 University of Illinois at Urbana-Champaign, Champaign, IL, USA

This manuscript describes the sequencing and assembly results for the (rather large, for a fly) genome of *Linnaemya tesselans*, a tachind fly. The genomic methods employed appear to be relatively state of the art, and a reasonable quality chromosomal level assembled genome has resulted. The genome has the potential to be highly useful for comparative studies of Diptera genomes, as it comes from a large and biologically interesting family of flies.

 The "Background" part of the manuscript presents a rather matter-of-fact outline of what is known of the fly, and that appears to be relatively little. It is not clear why this fly was chosen for genomic study. Does it represent a particularly interesting lineage of tachinids for comparative biological study? Given that it is not well known, how will the genome be used to leverage insights into the group and its biology? In other words, more detail is needed in the introduction so that users will have some idea how to effectively use the information in the paper. 

 Otherwise, it is a straightforward genome report with little to criticize.

Are sufficient details of methods and materials provided to allow replication by others?

Yes

Is the rationale for creating the dataset(s) clearly described?

No

Are the datasets clearly presented in a useable and accessible format?

Yes

Are the protocols appropriate and is the work technically sound?

Yes

Reviewer Expertise:

Systematics, ecology and evolution of parasitoid wasps, comparative genomics of wasps and their symbiotic viruses.

I confirm that I have read this submission and believe that I have an appropriate level of expertise to confirm that it is of an acceptable scientific standard.
